# A Study on the Current Situation of Prearranged Shelter Management in Japan for Making a Standard Operation Procedure

**DOI:** 10.3390/ijerph17249545

**Published:** 2020-12-20

**Authors:** Arisa Yasui, Muneyoshi Numada, Makoto Bando, Shintaro Nakano, Chaitanya Krishna

**Affiliations:** 1 Department of Civil Engineering, School of Engineering, The University of Tokyo, Tokyo 113-8654, Japan; 2 Interfaculty Initiative in Information Studies/Institute of Industrial Science, The University of Tokyo, Tokyo 113-8654, Japan; numa@iis.u-tokyo.ac.jp (M.N.); chaitugk@iitbombay.org (C.K.); 3 Division of Crisis Management Policy, Department of Crisis Management Environment, Tokushima Prefectural Office, Tokushima 770-8570, Japan; bandou_makoto_1@pref.tokushima.jp (M.B.); nakanoshintaro0301@gmail.com (S.N.)

**Keywords:** shelters management, designated shelters, residents participation, information sharing, local governments

## Abstract

To establish a standard operation procedure (SOP) for shelter management, this research analyzed current situations about shelter management in Japanese local governments, focusing on designation of shelters, operation manager and information sharing. The results indicate that some have non-designated shelters, which might lead to differences of support with designated shelters, local governments ask residents to operate shelters mainly, but this requirement is not shared among them, and system of information sharing is not developed primarily with the department of health and welfare. Besides, this research shows the importance of including the closing and after the closing of the shelters.

## 1. Introduction

Japan is a disaster-prone country. In recent years disasters have become more severe and frequent, such as the 2011 earthquake off the Pacific coast of Tohoku, the 2016 Kumamoto earthquake, and the 2018 heavy rainfall in western Japan. In a disaster, schools and public halls become shelters, and many residents evacuate to these locations [1,2]. Regarding these shelters, the Disaster Countermeasures Basic Law was amended in 2013, requiring the basic municipalities to designate shelters (Article 49-7 of the Disaster Countermeasures Basic Law), and consider evacuees in locations other than designated shelters. It stipulates (Article 86-7) that aspects of the living environment in shelters must be improve, such as the distribution of food, clothing, medicines and the provision of health care services (Article 86-6).

However, in the 2016 Kumamoto earthquake disaster, many evacuees evacuated outside the designated shelters, such as staying in the car, making it challenging to identify the evacuees and delaying support [3]. Also, in the event of a large-scale disaster, local government officials cannot take the lead in managing shelters from the perspective of a lack of human resources. On the other hand, the government’s “Guidelines for Ensuring a Good Living Environment in shelters” [4] recommends independent management by residents, but at present, there are few cases in which the discussion of division of roles in shelter management are thoroughly exhausted. Furthermore, if the department responsible for managing shelters and the department responsible for the health status of evacuees are separated within the local government, there is concern that health hazards may occur, such as delays in improving the shelter environment due to lack of cooperation. Based on this situation, it is necessary to review the designated shelters and determine the desired management entity and the health crisis management method for the victims.

Therefore, in this study, we focused on the status of prior arrangements regarding shelter management to examine the standard operation procedure (hereafter “SOP”) of a smooth shelter management system. The purpose is to understand designation of shelters (hereafter “DS”), operating entity and operation manager of shelters (hereafter “OEM”), and information sharing between the shelter and the disaster response headquarters/health and welfare department (hereafter “IS”). It is significant in that it provides suggestion for making advance preparation and a SOP of shelter management.

## 2. Literature Review

Many studies have clarified the actual situation of shelter operation from the viewpoint of a single municipality in each disaster and the case of a certain shelter [2,5,6]. However, with a limited approach to a certain shelter, there are many parts due to the peculiarities of the area [7], making it difficult to generalize. In addition, Ariyoshi et al. [8] clarified the status of the formulation and utilization of shelter management manuals based on a mail survey conducted nationwide. For example, the author of the manual, the status of a revision, the type of standard version/facility version, storage location, etc.

In recent years, it has been envisioned that shelters will be operated together with residents and support staff in addition to local government officials in the disaster area. In order for various leaders to jointly carry out smooth shelter operations, it is necessary to decide which shelter will be used (designation status of shelters), who and how shelters are operated (operation system) and how to share shelter information (information sharing), but a simple survey on these subjects is not sufficient. Also, the shelter management guidelines [9] of the Cabinet Office are composed of four perspectives, (Ⅰ) establishment of the shelter management system (normal time), (Ⅱ) operation of the shelter (after the disaster), (Ⅲ) response to needs, (Ⅳ) elimination of the shelter. Preparation is essential for smooth shelter operation, and there are a lot of tasks that must happen in regular times related to shelter operation. For example, the establishment of shelter operation system, the designation of a shelter, advance assumptions of initial concrete action, the establishment of a support system, measures for evacuees at home and people who have difficulty returning home. Also, the importance of information sharing such as the designation of shelters, the operation system of shelters, information management, and transmission is taken up as a checklist.

Therefore, in this paper, we first focus on DS, OEM, and IS, and organize past studies.

Firstly, during the disaster preparedness period, shelter location and evacuation routing operations are important. Researchers have proposed several models for shelter location and evacuation routing [10]. Kilci et al. [11] proposed a mixed integer linear programming-based methodology for selecting the location of temporary shelter sites and validated the model by using a city in Turkey as a case study. Bayram [12] classified previous research on evacuation planning and management models based on traffic assignment models. Overall, researchers have proposed several models for shelter location and evacuation routing: first as separate and then, more recently, as combined [10]. During the disaster response period, Haga et al. [13] clarified the characteristics of shelters for each facility type such as local community centers and elementary and junior high schools, and Sasaki et al. [14], investigated the actual use of temples as designated shelters. Also, since it is challenging to identify evacuees other than designated shelters such as undesignated shelters, necessary supplies and support may not be delivered [15]. In Mashiki Town at the time of the 2016 Kumamoto Earthquake, there was no function to integrate information on undesignated shelters, and information integration was delayed, and there was a lack of awareness of the occurrence of evacuees other than designated shelters and their support [16]. It was also clarified that the designated shelter had a function as a part of the support base in response to the request from the undesignated shelter. In addition, there was thought that it was necessary to dispatch local government officials to the designated shelter, and awareness that they could not respond due to the shortage of local government officials.

Next, the operating entity and operation manager of shelters will be described as examples of shelter operations in past disasters. Yamori [5] showed an example of a shelter in which the operating system was gradually changed from time to time while utilizing volunteers under the strong regional leader in the Great Hanshin-Awaji earthquake. He also emphasized the importance of the evacuees themselves being involved in the operation for the evacuees’ independence. After the Niigata Chuetsu-Oki earthquake, the local community was living in an evacuated situation while operating the shelter independently [17]. Kobayashi et al. [6] clarified three groups of residents responsible for voluntary management: local leaders, community activity leaders, and circle activity participants. It was shown that inter-organizational cooperation based on the existing organization is necessary. In the Great East Japan earthquake, all teachers played a central role in the shelter located at the Ishinomaki City Hebita Elementary School. Also, the teachers took command of the operation of the shelter, and the principal made various decisions [2]. The shelter operation manual in the “Miyagi Disaster Prevention Education Basic Policy” [18] states, “Although the operation of shelters is the business of the municipalities, there are many cases where cooperation is required even on the side of providing facilities, so be familiar with the contents”. However, when the degree of damage was extensive and a wide area was damaged, such as the Great East Japan earthquake, the functions of municipalities were confused and the situation assumed by this manual was not reached.

Finally, regarding information sharing between other departments, one of the lessons learned from the Great East Japan earthquake was that it was not possible to grasp shelter and evacuees information, and to take effective measures based on appropriate situational awareness [19]. Bando et al. [20] stated that it is necessary to share information between the disaster prevention department of the local government and other departments/within each department, in order to uniformly grasp the shelter situation for realizing effective response. In addition, although information sharing within departments has improved, the current situation is that information from other departments is not being distributed across the board.

As described above, regarding DS, there are studies on the intention to use undesignated shelters that occur after a disaster. However, the study about undesignated shelters that the local government grasps in advance is not enough. Regarding OEM, the state of operation by various entities, and the elements necessary for the operation by a non-local government are clarified from actual individual cases. However, the national trend of cognitive sharing is not clear. Regarding IS, the necessity of information sharing has been shown, but few studies are focusing on shelters.

Based on the above, considering that preparation is vital for smooth shelter operation, there is novelty in that we focused on the status of three advance arrangements regarding shelter operation, DS, OEM, and IS. These results are useful in order to make a standard operation procedure of shelter management because they provide suggestions from the perspectives of designating shelters, examining the operating entity and operation manager, and establishing an information-sharing system. As a method of this research, we use a questionnaire survey of local governments’ disaster prevention departments nationwide.

## 3. Methods

This research was jointly conducted by the University of Tokyo Institute of Industrial Science and Tokushima Prefecture. The target of this study is the department in charge of disaster prevention in local governments nationwide. Table 1 shows the outline of the survey conducted. The number of valid responses was 420.

**Figure 1:**
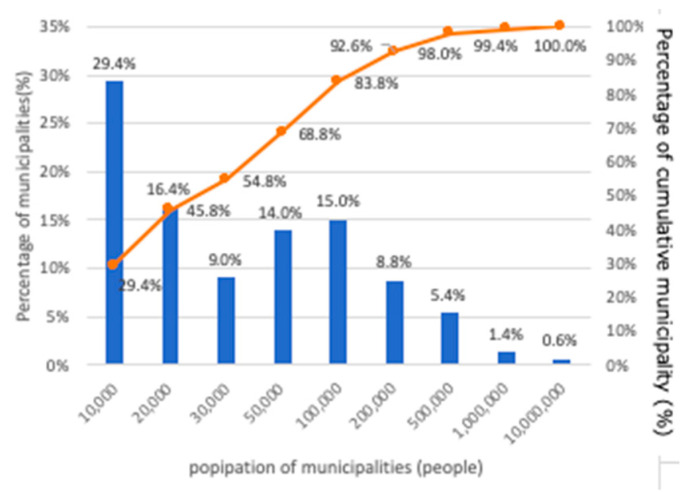
Population scale by municipality nationwide.

**Figure 2:**
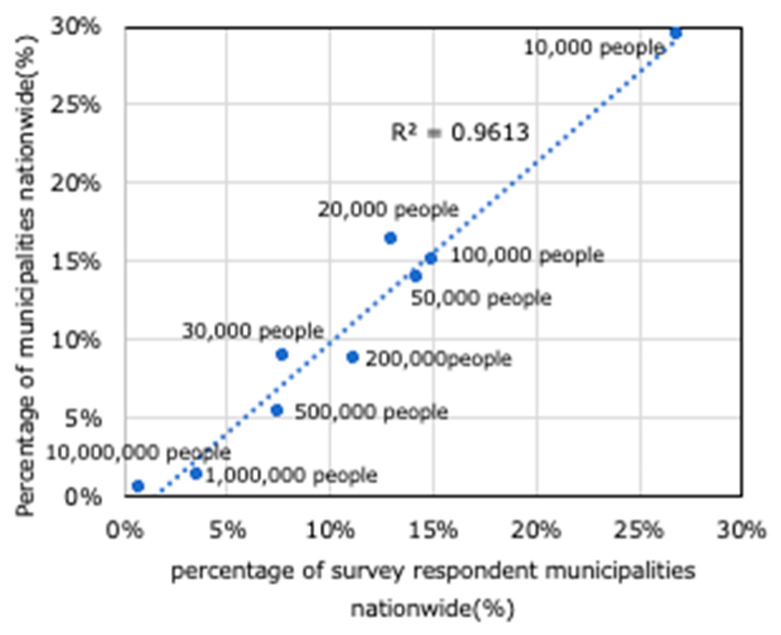
Population size by municipality and percentage of survey respondent municipalities nationwide.

Figure 1 shows the population size by the municipality nationwide (national census, 2015 survey). According to this, it can be seen that 29.4% of all 1741 municipalities have a population size of less than 10,000, and 68.8% have a population of 50,000 or less. In contrast, as shown in Figure 2, the population size by municipality and percentage of survey respondent municipalities nationwide show similar trends. It can be understood then that the results obtained in this paper reflect a national trend.

**Figure 3:**
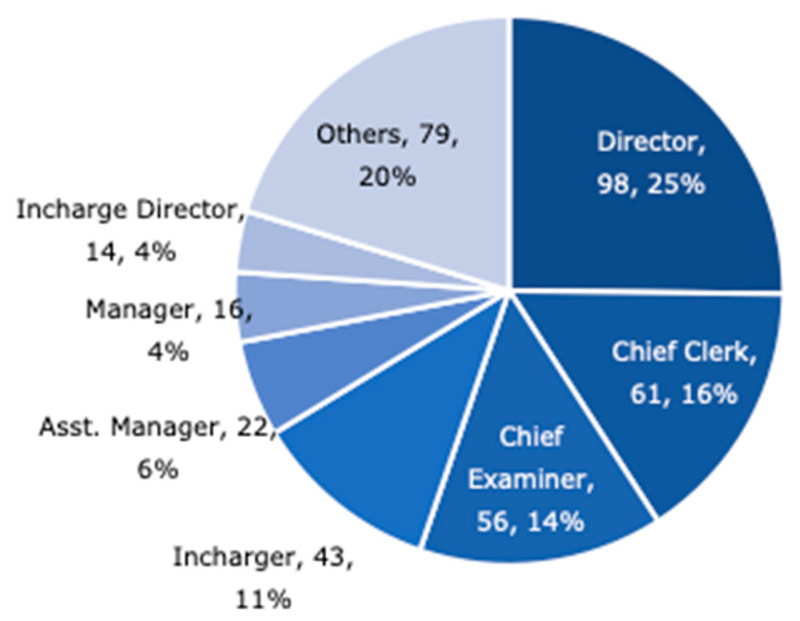
Job title ratio of survey respondents.

Figure 3 shows the rank of the staff member who submitted the response. Directors, chief clarks and chief examiners account for more than half of the respondents. The chief respondent is the most common answer, but it is also positioned as the contact person for this survey. It cannot be said that the chief has prepared the answer.

**Figure 4:**
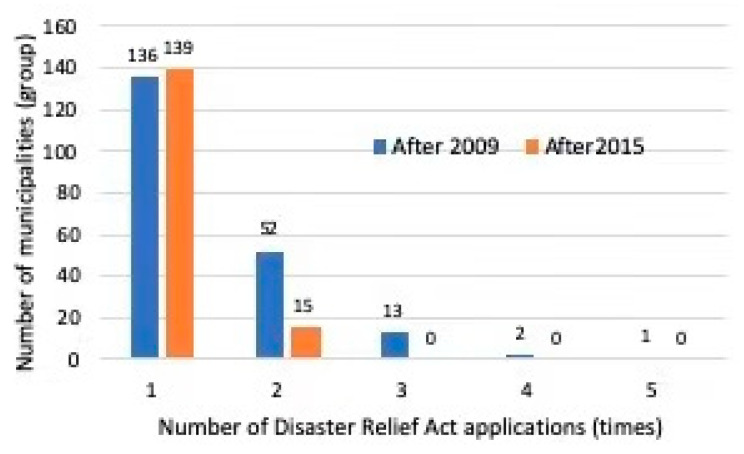
Presence or absence of disaster experience.

The disaster experience is defined as whether or not the Disaster Relief Act was applied. Here, the Disaster Relief Act’s application status announced by the Cabinet Office is targeted from 2009 to 2015 before this survey. As shown in Figure 4, the number of responding municipalities for which the Disaster Relief Act was applied even once is 204 (about 49% of all responses, 204/413), and there are 136 responding municipalities applied once and one municipality applied it five times, respectively. In addition, the number of municipalities to which the Disaster Relief Act has been applied since 2015 is 154 (about 37% of all responses, 154/413). Comparing after 2009 and after 2015, the number of local governments that applied it one time wass higher after 2015 than after 2019. This is because the number of times is reduced to one when totaled after 2015.

Table 2 shows the composition of the questionnaire. Most of them are selective, but some questions are descriptive. Selective answers is the format to has three or more answer categories for a question and has one or more answers selected from among them. On the other hand, the descriptive answer is the format where respondents are free to write. We use descriptive answers when asking about issues based on actual experience because it was assumed that the contents of the answers would be diverse.

The structure of this study is as follows: Section 4 shows the results of prior arrangements regarding shelter operations. The results of DS, OEM, and IS are shown. In addition, issues based on experience will be described for each business process of shelter management. Finally, in Section 5, based on the clarified issues, the direction of the SOP of the shelter management system is considered from the viewpoint ofDS, OEM, and IS.

## 4. Status of Prior Arrangements Regarding Shelter Management

### 4.1. Designation of Shelter

**Figure 5:**
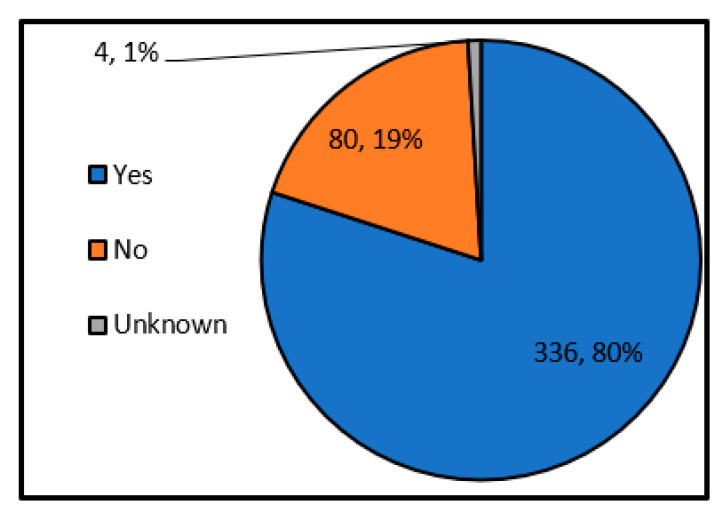
Presence or absence of undesignated shelters (single answer) (Single answer, total number of answers 420, total number of local governments: 420, unit: group).

First, we investigated the situation of undesignated shelters. Here, an undesignated shelter is defined in the question text as “the shelter that is not designated based on the Disaster Countermeasures Basic Law, but the residents are informed by hazard maps, etc.”. Also, the designated emergency shelter is excluded. For example, evacuation meeting places (primary evacuation places) of self-government (ward) associations, private educational facilities, welfare facilities, etc. Figure 5 shows the results of a survey on the existence of undesignated shelters. According to this, it was found that about 20% of local governments have undesignated shelters. In addition, according to the descriptive free answer, there are problems like “I could not grasp the whole of private and private shelters” and “In many cases, I could not contact the facility manager at undesignated shelters”.

In the 2015 White Paper on Disaster Prevention [21], there is no clear distinction between shelters for avoiding the danger of imminent disasters in the Great East Japan earthquake and shelters for subsequent evacuation life, and the designation of shelters for each disaster is specified. It has been pointed out that some victims were struck by the tsunami even though they fled to the evacuation site because they had not been evacuated. Therefore, the Disaster Countermeasures Basic Law was revised to judge facilities and terrain comprehensively. It is obligatory to secure designated emergency shelters for each disaster and designated shelters for temporary evacuees. From this situation, the reason of the existence of undesignated shelters is considered the shortage of data, personnel, and time for confirming the safety of each disaster type. In the wake of the Great East Japan earthquake, the assumptions of all hazards have been revised nationwide, and it is necessary to use the new information when designating shelters. It is possible that the review has not been completed yet, or that alternatives to facilities that cannot be used as shelters are under consideration based on new assumptions. In addition, since it is necessary to consider the type of disaster, the local governments where there is a high risk of various disasters, it may take time to consider.

Such undesignated shelters have not been confirmed to be safe in the event of a disaster. As Araki et al. [16] points out, there is a disparity in support between designated shelters and undesignated shelters. It is suggested that it is necessary to proceed with the designation of the place.

### 4.2. Operating Entity and Operation Manager of Shelters

#### 4.2.1. Shelter Operating Entity

**Figure 6:**
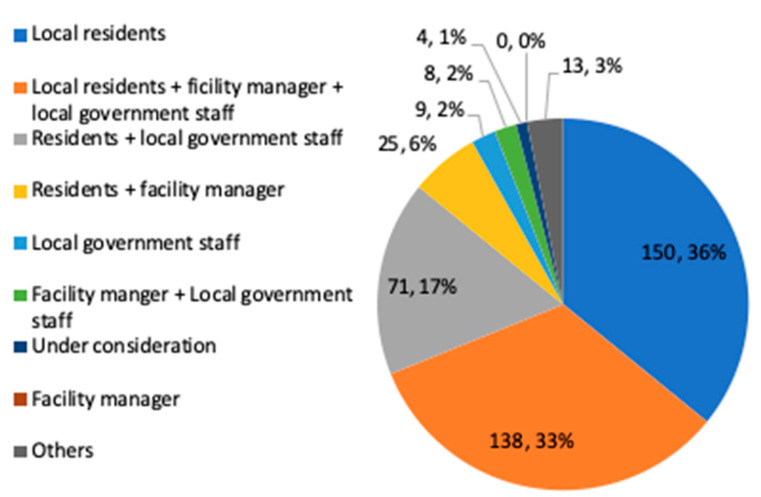
Shelter operating entity (single answer) (Single answer, total number of answers 418, total number of local governments: 418, unit: group).

Figure 6 shows the shelter operating entity assumed by the local government. As a result, it was found that the number of local governments that think that it is desirable to have shelters operated mainly by local residents is almost the same as the number of local governments that think that it is desirable to operate shelters by local residents + local government officials + facility managers, with a ratio of about 30%. The Cabinet Office recommends that municipalities, facility managers, and residents’ organizations cooperate with each other [4], but according to this result, it can be said that this cooperation isn’t enough. Also, from the descriptive free answers, regarding the operation led by local residents, there are the problems of “it is difficult for residents to judge whether the shelter are safe” and “Establishing the management of the keys when voluntary disaster prevention organization open shelters”. Regarding the operation led by the cooperation with the facility managers, it became clear that problems had occur such as “It took time to contact the facility manager when opening in the early morning” and “Because the cooperation system between the city and the school was not clear (especially the criteria for gathering teachers, handling public affairs related to engaging in shelter operations, handling overtime work orders, and burdening costs such as security), it took time to coordinate with the designated manager”.

The operation led by local residents is useful in compensating for the lack of human resources of local government officials and independence for reconstruction 5, but it is necessary to consider that problems may occur due to lack of specialized knowledge. When collaborating with facility managers, it is thought that troubles in the event of a disaster can be prevented by making arrangements in advance. Also, as Ariyoshi et al. [8] point out, to prevent confusion of shelter management by residents, it is convenient that the manual is already installed in the shelter at the time of opening. The manual is also considered to be useful for communicating knowledge to residents.

In addition, no local government answered that only the facility manager was the shelter operating entity. The reasons for this are as follows. First, there are cases of shelter management by school staff in past disasters [2], but there is a high possibility that the local government does not think the situation is good. Also, regarding shelter operation entity, Article 56 states “Head of designated administrative organs and designated local administrative organs, heads of local public organizations and other executive organs, designated public institutions and designated local public institutions, and facility managers important for disaster prevention”. Since the facility manager is described in the end, it is unlikely that it is the operating entity.

**Figure 7:**
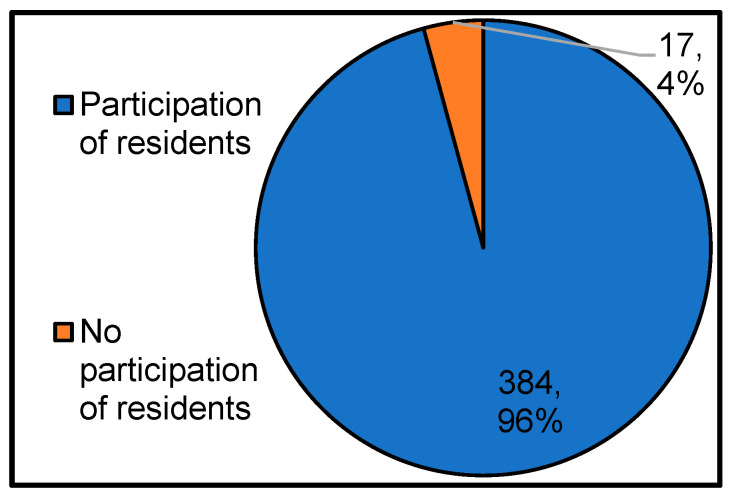
Presence or absence of participation of local residents, who are the shelter operating entities. Fix broken words in figure labels

Figure 7 shows whether residents are expected to participate in the shelter operating entity. As a result, it can be seen that more than 90% of local governments expect the participation of local residents. It is assumed that local residents participate because the idea of voluntary management by residents recommended by the government [4] is widespread in local governments nationwide, and there are limits to the operation of shelters by the administration alone due to many disasters.

**Figure 8:**
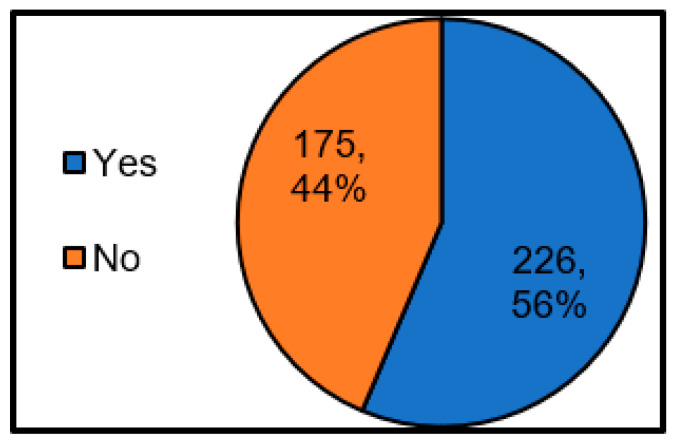
Presence or absence of involvement of local government officials who are the shelter operating entities.

Figure 8 shows whether the shelter operating entity includes local government officials. As a result, it is assumed that more than half of the local governments will be involved in the operation of shelters. It can be said that local government officials are desirable to be the operating entity because as a general rule, municipalities are required to secure shelters (Article 49-7 of the Disaster Countermeasures Basic Act) and strive to maintain a suitable living environment for evacuees (Article 86-6). On the other hand, in the descriptive free answer, “There is a limit to the operation of shelters by local government officials, and it is ideal to operate shelters by local residents such as voluntary disaster prevention organizations.” and “In current situation, local government officials operate shelters. However, it is becoming challenging due to the shortage of staff. We would like to establish a system so that local residents can operate it in the future. “It can be said that there is an idea that it is desirable not to regard the local government officials as the management entity but to devote them to other duties because of the shortage of local government officials, which is one of the major problems in the operation of shelters often pointed out [22].

**Figure 9:**
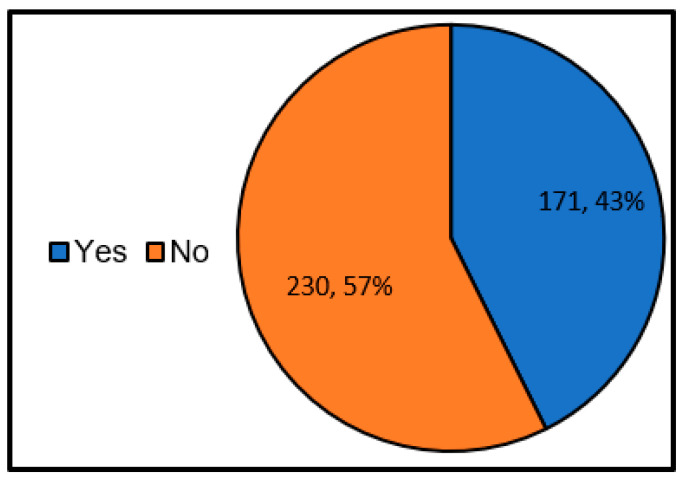
Presence or absence of participation of facility managers, who are the shelter operating entities.

Figure 9 shows whether the shelter operating entity includes facility managers. As a result, it was found that less than half of the local governments included facility managers as shelter operating entities. In the descriptive free answer, there was an opinion that “there may be no knowledge about the facility without the facility manager.” Therefore, it is thought that the operation work will be more efficient with the facility manager’s involvement who is familiar with the facility.

**Figure 10:**
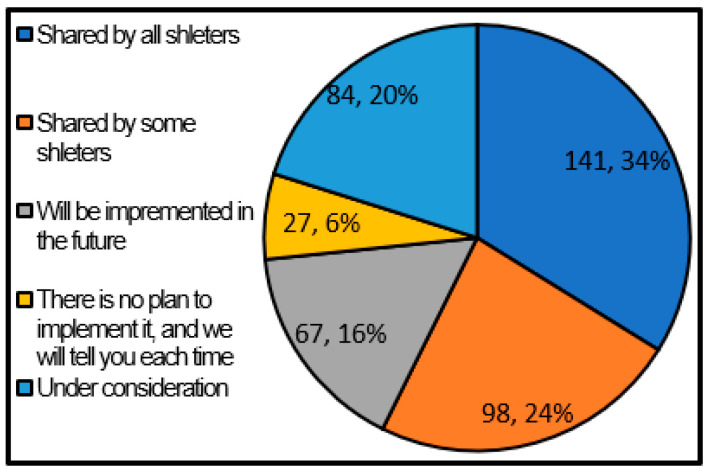
Sharing of awareness about the shelter operating entity (single answer) (Single answer, total number of answers 417, total number of local governments: 417, unit: group).

Figure 10 shows whether or not the local government’s concept is made known to the concerned parties (local residents, facility managers, etc.), and the recognition is shared regarding the shelter operating entity. As a result, 34% of the local governments answered that they shared their awareness of all shelters, and 24% of the local governments that answered that they shared their awareness of some shelters. Therefore, the local governments that share recognition with even some shelters was about half (58%). It became clear that this is different from the fact that more than 90% of local governments are assuming the participation of local residents as the operating entity. As a method of communicating the recognition of the municipality to the residents, it is conceivable to create a manual of shelter management in cooperation with the residents. However, according to Ariyoshi et al. [8], the rate of creating manuals in collaboration with residents is low, and it can be said that many local governments have not been able to disseminate the recognition and knowledge necessary for independent shelter management to residents through the creation. This study is consistent with this. In addition, from the local governments that have actually opened shelters, there were opinions such as “too few residents are aware that they are the operating entity of shelters.”, “Evacuees think shelter management are all administrative work. (Lack of self-help)”, and “The evacuees themselves recognize that they are customers”. These are considered to be due to the lack of sharing of awareness regarding the shelter operating entity.

#### 4.2.2. Shelter Operation Manager

**Figure 11:**
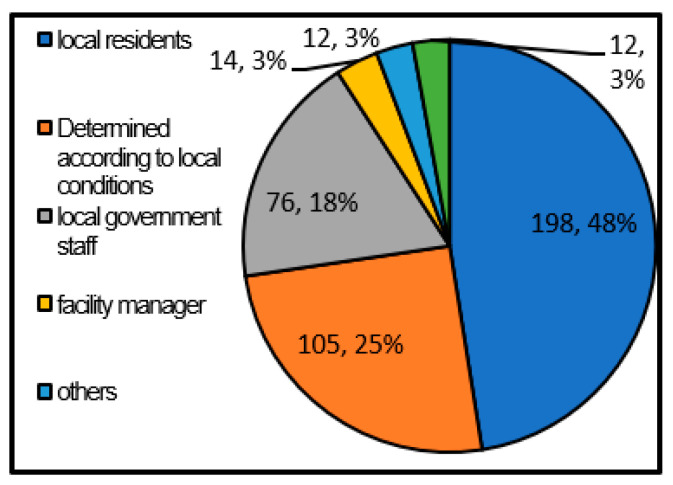
Shelter operation manager (single answer) (Single answer, total number of answers 417, total number of local governments: 417, unit: group).

Figure 11 shows the shelter operation manager assumed by the local government. As a result, it was found that many local governments think that the representatives of residents are desirable as the managers of shelters.

Also, 1/4 of the local governments answered that they would choose from representatives of residents, facility managers, and local government officials according to the local situation. It is good to decide according to the situation of the area instead of assigning a unified manager because they can use each area’s characteristics. However, since it is not decided, it may not be possible to operate the shelter smoothly in the event of a disaster.

**Figure 12:**
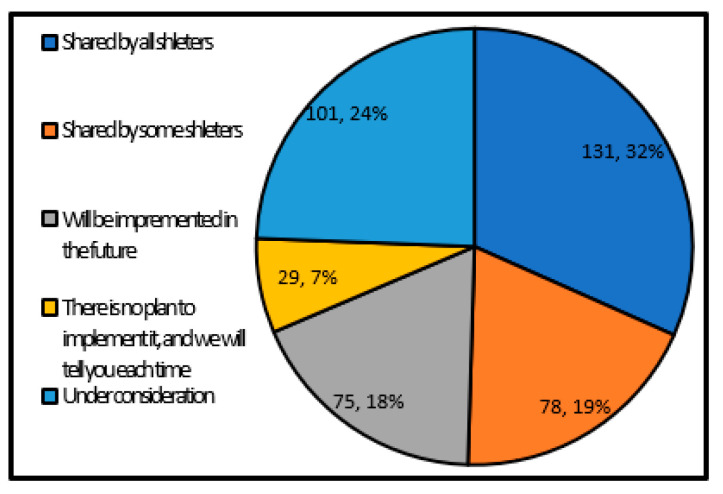
Sharing the awareness of the shelter operation manager (single answer) (Single answer, total number of answers 414, total number of local governments: 414, unit: group).

Figure 12 shows the degree to which the local government’s thought of operation manager is recognized and shared. As a result, compared to the shelter operating entity’s shared awareness, about 1/3 shared the awareness among all the shelters and about 1/4 are under consideration. Although the overall tendency is similar, the number of local governments that share the recognition of the shelter operation manager is decreasing. It can be said that the shelter operation manager has not been examined as compared with the shelter operating entity.

### 4.3. Information Sharing at Shelters

#### 4.3.1. Information Sharing System with the Disaster Response Headquarters

**Figure 13:**
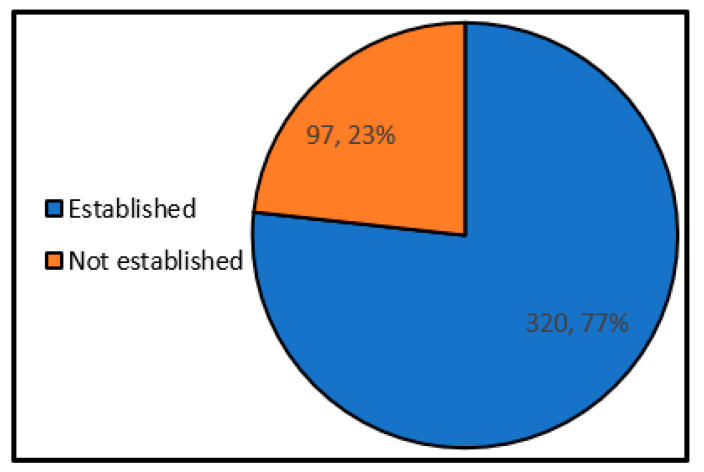
Information sharing system rules with disaster response headquarters (single answer) (Single answer, total number of answers 417, total number of local governments: 417, unit: group).

Figure 13 shows the status of information sharing with the disaster response headquarters. As a result, 1/4 of the local governments do not have rules for sharing information with the disaster response headquarters. From the descriptive free answers such as “There was a problem in sharing information such as the number of evacuees accepted and supplies between the disaster response headquarters (disaster prevention department) and each shelter.”, and “We weren’t able to share information between the shelter and the disaster response headquarters”, it became clear that there are many issues of information sharing in local governments that have experience of operating shelters. It is considered to be necessary to establish an information-sharing system as a preliminary agreement.

**Figure 14:**
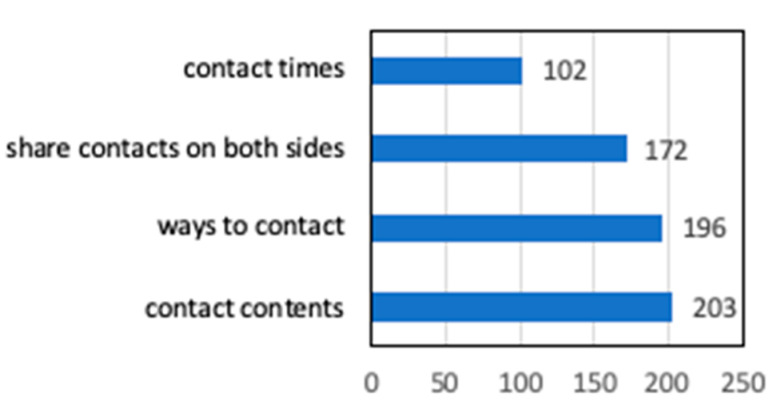
Information sharing with the disaster response headquarters (multiple answers) (Multiple answers, total number of responses 770, total number of respondents: 417, unit: number of responses).

Figure 14 shows the contents of the information-sharing system. Of the local governments that specify information sharing with the disaster response headquarters, the number of the local governments that specify the contact times (at the time of opening/morning/evening, etc.) are less than that of the local governments that set the ways to contact and contact contents and share contacts to both sides. For information sharing, most local governments create assessment sheets of shelters in disaster prevention plan or manuals for shelters [23,24], but it is likely that the contents of assessment sheets are not enough.

On the other hand, even if the means of communication was set, it became clear from the descriptive free answers that there are various problems such as “Many local government officials were not accustomed to handling wireless communication as a means of communication with each shelter and could not convey information smoothly.”, “I could not contact the disaster countermeasures headquarters because I could not use the system disaster prevention administrative radio”, and “There is a communication error due to sensitivity, and there is a cross line when using wireless information communication”.

**Figure 15:**
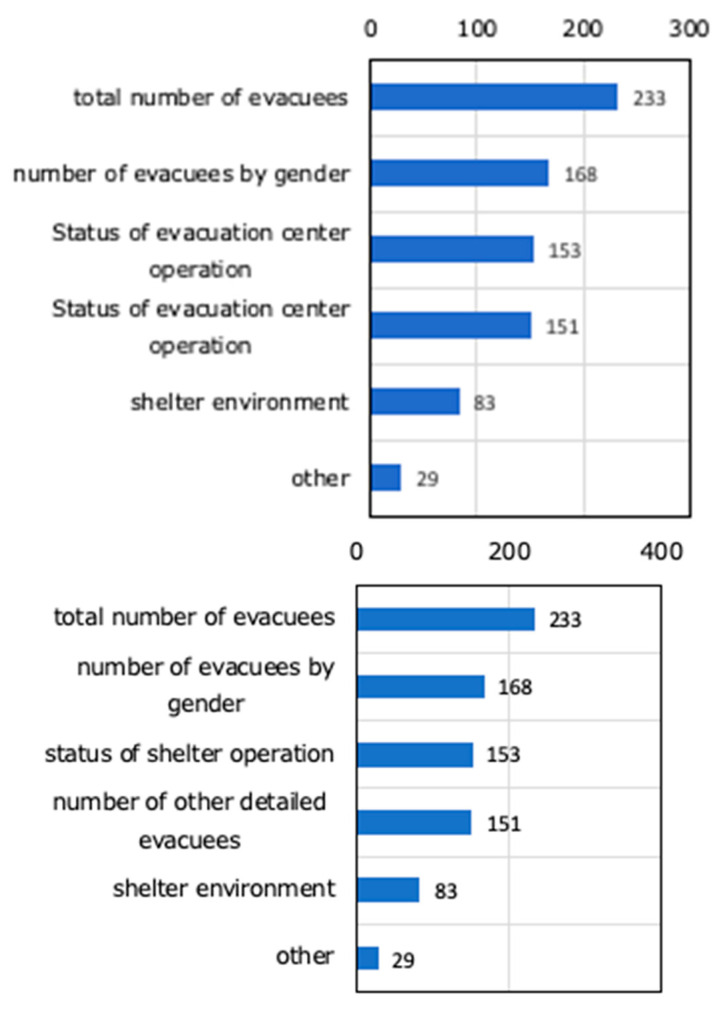
Contents of information sharing with the disaster response headquarters (multiple answers) (Multiple answers, total number of responses 814, total number of respondents: 203, unit: number of responses).

Figure 15 shows the contents of information sharing with the disaster response headquarters. According to this, it was found that the total number of evacuees was large but some local governments do not provide detailed information on the number of evacuees, such as gender, older people, children, and pregnant women. It was also found that few local governments have stipulated to inform the shelter environment (hot/cold/population density/number of toilets/number of beds). Since sharing information on this shelter assessment leads to maintaining a suitable living environment for evacuees, it is considered necessary to analyze what kind of information items would contribute to maintaining a suitable living environment for evacuees and reflect this on the shelter assessment sheet.

Other listed contents are shown below:

- Damage situation of the shelter
- Surrounding conditions (lifelines, collapsed houses, road conditions, fire conditions, human damage)
- Number of injured and sick
- Necessary supplies/equipment/food/human resources
- Requests from evacuees
- Gathered staff, facility managers, voluntary disaster prevention organization

It turned out that while some local governments set specific information contents sharing with the disaster response headquarters, other local governments do not, as shown in Figure 10, and there are major differences between them. This is thought to be due to differences in the preparation status due to differences in the number of staff (human resources) and other circumstances in each local government. Also, considering that many of the support staff are dispatched to shelters, it is thought that adequate support will be achieved by establishing information-sharing systems.

#### 4.3.2. Information Sharing with Health and Welfare Departments

**Figure 16:**
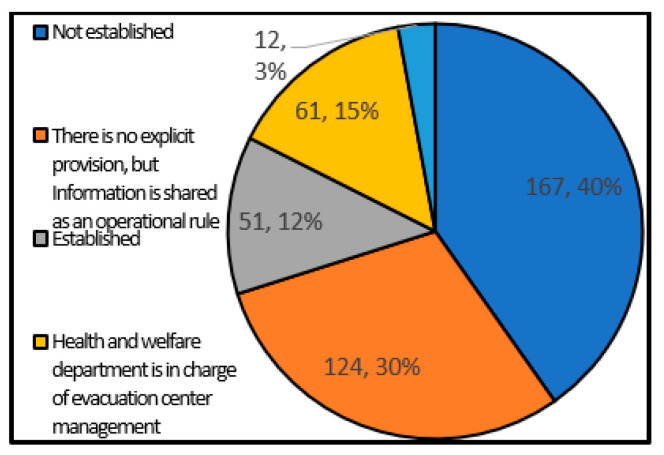
Information sharing with health and welfare departments (single answer) (single answer, total number of answers 415, total number of local governments: 415, unit: group).

Figure 16 shows the status of information sharing with the health and welfare department. As a result, there are some local governments have centralized information because the health and welfare department is in charge of shelter management. Comparing with Figure 10, about 100 local governments did not stipulate information sharing with the disaster response headquarters (Figure 10), more than 150 local governments have not stipulated information sharing with health and welfare departments, indicating that the information-sharing system has not been developed. Since cooperation with health and welfare departments such as shelter assessment and supply of supplies is considered to be essential for maintaining the health of disaster victims, it can be said that there is an urgent need to develop an information-sharing system.

### 4.4. Issues Related to Shelter Management

It is stated that the issues related to shelter management were clarified from the results of the descriptive free answer of this survey. The descriptions of the obtained free answers were grouped according to the similarity of the contents, and they were classified into four points: when the shelter is opened, at the time of acceptance, closing stage, and overall management, from the viewpoint of the business process of shelter operation. Table 3 summarizes the results.

#### 4.4.1. When the Shelter Is Opened

The issues at the time of opening a shelter were divided into four points: dealing with evacuees in advance, informing residents of the opening of a shelter, gathering staff in charge of opening, and the key. First, if there is no need to evacuate to a shelter or if a shelter has not been opened at an early stage, it is difficult to deal with it because there may be evacuees in advance. Secondly, the shelters that were opened were not well known, and residents evacuated to shelters that had not been opened, and the evacuees returned home because the display of the shelters that had been opened was unclear. Thirdly, if the gathering of local government officials and school faculty members in charge of the opening is delayed, the shelter’s opening will be delayed. If the gathering is not possible, the shelter cannot be used. Fourth, it may not be possible to unlock the key due to the key’s loss or change. The problem is that the keys cannot be delivered well.

#### 4.4.2. At the Time of Acceptance

When accepting evacuees, the issues were divided into four points: people with mobility difficulties, many evacuees, pets, and people requiring special attention. First, some people have difficulty moving to the shelter, such as the elderly and those who need long-term care, and it is an issue to secure transportation between their homes and the shelter for such people. Second, when a large number of evacuees evacuate at the same time, they may be in a state of confusion and cannot be accepted well. If many evacuees are concentrated in one place, they may not be accepted if the capacity of the shelter is exceeded. Third, it was found that about 20 local governments have problems in accepting pets. For example, responses to evacuees who wish to evacuate with their pets, consideration of the location of cages, etc. Fourth, there are problems of responding to people requiring special attention and communication with foreign evacuees despite a shortage of personnel.

#### 4.4.3. Closing Stage

The issues at the closing stage of the shelter were divided into four points: responding to evacuees returning home before closing, the occurrence of residents refusing to leave, closing at night, and cleaning up.

**Figure 17:**
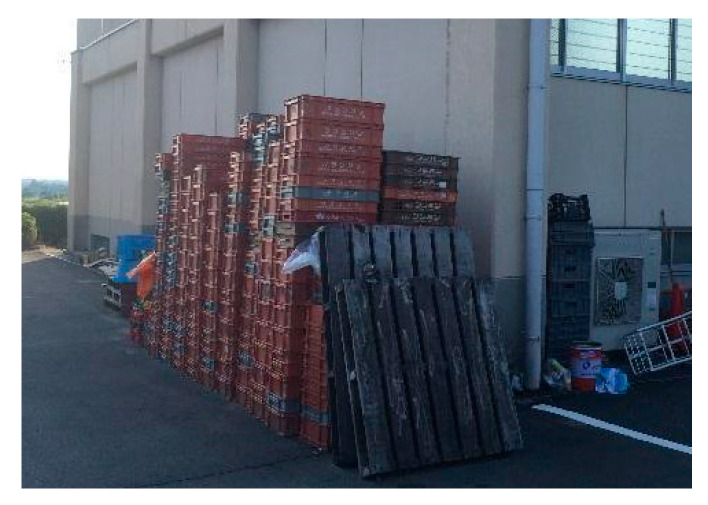
Nishihara Village Office during the 2016 Kumamoto Earthquake. Uncollected pallets (photographed by Numada).

First, residents may wish to return home from the shelter in a situation where there is still a risk of disaster, and the evacuees may return home without contact. Second, although it was decided to close the shelter, some residents may not return home due to convenience and anxiety, and it may be difficult to deal with. Thirdly, when closing at night, special measures may be required, such as the fact that the damage situation cannot be confirmed and the closure cannot be performed, and that staff members wait overnight for evacuees who do not return home. Fourth, there are issues such as processing after using and storage of stockpiles and a shortage of personnel for tidying up work. Although the pallets were provided to Nishihara Village during the 2016 Kumamoto Earthquake, the pallets were not collected after that and uncollected pallets were piled up (Figure 17), forcing local government officials to process them.

#### 4.4.4. Overall Management

The overall management issues were divided into three points: dealing with residents who lacked self-help awareness, long-term management system, and maintaining the health of evacuees.

First, it was confirmed that many residents recognize that the operation of shelters is the role of public assistance. Therefore, the issue is how to raise awareness about self-help at shelters. Second, the shortage of substitutes for long-term operation and the increase in shelter staff burden will be issues. Thirdly, it is needed to take measures against chronic and infectious disease of evacuees. In order to do this, it is necessary to establish an information-sharing system with shelters, but as shown in this paper, the system remains undeveloped in many local governments at present.

As mentioned above, the issues in the four business processes have been clarified. In particular, the management and delivery of keys for shelters, the response to residents and tidying up due to closure, etc. are not described in the Cabinet Office’s shelter management guidelines 17). Therefore, it can be said these should be added as a new perspective. There are few alternatives when the key is not at hand, and it is not a problem that can be tackled with the cooperation of many people. Although it should be avoided from the viewpoint of security, it is necessary to establish rules among the parties concerned. Regarding the response to residents due to the closure, it is expected that the demand for highly urgent work and a sense of speed will decrease at the closing stage, but both the evacuees and the operating entity are physically and mentally tired, and problems that do not usually occur can occur. It is difficult to imagine such trouble in peacetime, but a real problem can be grasped by referring to actual cases and considering that the mental state is different. It can be said that tidying up after closure is returning to the normal state and starting countermeasures for the next disaster. Preparation is crucial in disaster countermeasures, and it is desirable to pay attention to post-disaster work in the sense that it corresponds to the preparation.

## 5. Conclusions

The purpose of this study is to clarify the local governments’ current situation of undesignated shelters, operating entity and operation manager of shelters and their recognition sharing, and the information sharing system between the shelter and the disaster response headquarters/health and welfare department, to create a SOP in Japan.

As a result, although there is a study by Ariyoshi et al. [8] as a nationwide survey on shelter management, the following results were obtained in this paper compared to these related studies in the past.

Regarding the designation of shelters, it is necessary to regularly check the facilities and disaster safety of existing designated shelters. On the other hand, in undesignated shelters, safety in the event of a disaster has not been confirmed. There is a disparity in support between designated and undesignated shelters, so it is highly necessary to proceed with shelters’ designation.

Most local governments assume residents’ participation regarding the shelter operating entity and the operation manager. However, it can be said that the idea is not sufficiently known to the residents. It became clear that local government officials felt that many evacuees lacked self-help consciousness. In the future, it will be necessary to study how to describe it in regional disaster prevention plans and district disaster prevention plans and to promote public awareness and sharing with residents.

Regarding information sharing at shelters, about 1/4 of the local governments do not have a communication system with the disaster response headquarters. Also, the information sharing system with the health and welfare field has not progressed any further. It is necessary to establish clear rules and repeatedly examine the effectiveness of the rules, such as whether the defined information sharing means will function in a disaster.

In addition to the above three points, the items and issues to be considered were clarified by dividing them into the opening, acceptance, closing, and overall shelter management. In particular, the management and delivery of shelter keys, the response to residents, and tidying up due to closure are not described in the shelter management guidelines [9] of the Cabinet Office, so it can be said that these should be added as a new perspective.

It is also essential to take measures against infectious diseases in the operation of shelters, such as the state of emergency being issued on 7 April 2020, following the spread of the new coronavirus. Even at present, the response to infectious diseases is stored in a database, but the content is insufficient, so we would like to enhance this.

## Figures and Tables

**Table 1:** Outline of the survey.

Items	Contents
Name of the survey	Survey on shelter management
The target of the survey	Disaster prevention departments of local governments nationwide (including special wards)
Contents of the survey	Shelter management policy etc.
Survey period	From mid-December 2019 to mid-February 2020
Collection method	Send a Google form or word file to the municipality whose email address was published on the website
Valid responses	420/1100 shipments (38% recovery rate). Of the 420, there were seven answers from prefectures, and the ones that corresponded to the prefecture of the question were answered.

**Table 2:** The structure of the survey.

Classification	Answer Format	Main Question Items
Designation of shelters	Selective single answer	Number of designated shelters Number of undesignated shelters
Shelter management	Selective single answer	Operating entity Operation manager Operation manual
Information Sharing	Selective single answer Selective multiple answers	Contact with the disaster response headquarters Contact with health and welfare departments
Issues based on actual experience	Descriptive free answer	When opening, accepting or closing a shelter Task (descriptive formula)

**Table 3:** Issues related to shelter management.

Work Process	Classification	Issues
When the shelter is opened	To pre-evacuees Correspondence	Voluntary evacuees occurred at an early stage It is difficult to respond because shelters may not be opened.
	Dissemination of shelter establishment to residents	If all shelters are not opened, facilities that the citizens think are opened will not be opened, and the citizens will be confused. The shelter assumed in the district and the shelter established by the city are different. Since the shelter opening display is unclear, some citizens go home without knowing
	Gathering of establishment staff	The arrival of staff may be delayed and the opening may be delayed.
		The facility manager (school teacher) was not present and there is a part where the school building cannot be opened.
	Keys	The key to the gate has changed and cannot be unlocked. The key to the disaster prevention warehouse is lost. Key delivery from the facility manager is not smooth.
At the time of acceptance	Persons who have difficulty moving	Persons requiring special care who have difficulty moving to designated shelters (elderly and single). It is necessary to secure transportation (pick-up, etc.) between evacuees’ home and the shelter.
	Many evacuees	More and more evacuees are coming and getting confused, and we can’t accept them well. There will be a flood of evacuees than expected, resulting in unacceptable situations.
	pets animal	There was a request to accompany the shelter. It is difficult to deal with it on site. Outdoors are appropriate when allergies are taken into consideration. However, due to heavy rain and strong winds, it is difficult to deal with it.
	People in need	Response to a person requiring support evacuates. There is a problem with how to communicate with foreign evacuees.
Closing stage	Response to evacuees returning home before closure	Responding to evacuees who have offered to return home when safety has not been confirmed. Responses such as searching when evacuees return home without saying anything before closing.
	The occurrence of residents refusing to leave	Although it was decided to close it, some residents did not return home and it was difficult to deal with.
	Closing at night	The damage situation cannot be confirmed and it cannot be closed. Even if the danger of a disaster has passed, some residents don’t want to return home and a staff has to be on standby overnight.
	Cleaning up	It is difficult for the staff in charge of shelters alone to clean up used mats, blankets, dust, etc. If the disposal method (collection, disposal, etc.) of the stockpile used at the shelter doesn’t decided, it will be left in the facility for a while.
Overall management	Responding to residents who lack a sense of self-help	Dependence on local government officials. Residents depend on the administration. As a result of the management being carried out by the administrative body, the degree of dependence on the government has increased and residents’ troubles or demands escalate. There are too many requests from evacuees for stocked blankets, rugs, slippers etc.
	Long-term management system	There will be a shortage of substitutes over a few days. The burden on shelter establishment staff (even if a shift system is in place) increases.
	Maintaining the health of evacuees	It is necessary to understand the pre-existing illness and isolate it when influenza occurs. Correspondence to new coronavirus.

## References

[1] Ueyama K., Saito K., Matsumoto Y., Yahiro S., Yamamoto R., Kurokawa T. (2018). Current status and future suggestions of Niimi University as a shelter due to the heavy rain disaster in July 2018. Bull. Niimi Univ..

[2] Ito T., Kawana H. (2016). School as a “designated shelter” in the event of a disaster A case study at an elementary school located in an area affected by the Great East Japan Earthquake. Ibaraki Univ. Fac. Educ. Bull..

[3] Mainichi Newspaper: Kumamoto Earthquake Support, the Difference in Shelters, No Food Supply in the Surrounding Area, Medical Team Resident in the Central Area. 22 April 2016. https://mainichi.jp/articles/20160422/ddm/003/040/064000c.

[4] Cabinet Office (2013). Guidelines for Ensuring a Good Living Environment in Shelters.

[5] Yamori K. (1997). Shelter management in the Great Hanshin Earthquake-The step-by-step transformation process. Jpn. Soc. Psychol. Exp..

[6] Kobayashi H., Ichiko K., Nakabayashi K. (2010). Consideration on the possibility of shelter management centered on the local community-A case study of the Hisumi district of Kashiwazaki City during the 2007 Niigata Chuetsu-oki Earthquake. Proc. Soc. Reg. Saf..

[7] Kondo T., Koshiyama K., Benitani S., Kondo S., Mizunaka S. (2008). Research on cross-organizational system and command and coordination function of disaster response headquarters—A case study of Niigata prefecture in the Niigata Chuetsu-oki Earthquake (2007). Proc. Soc. Reg. Saf..

[8] Ariyoshi K., Shibano M., Sasaki S. (2020). Research on the creation and utilization of the “shelter Operation Manual”—Based on the National Local Government Mail Survey. Proc. Soc. Reg. Saf. Stud..

[9] Cabinet Office (2016). Shelter Management Guidelines.

[10] Amideo A.E., Scaparra M.P., Kotiadis K. (2019). Optimising shelter location and evacuation routing operations: The critical issues. Eur. J. Oper. Res..

[11] Kılcı F., Kara B.Y., Bozkaya B. (2015). Locating temporary shelter areas after an earthquake: A case for Turkey. Eur. J. Oper. Res..

[12] Bayram V. (2016). Optimization models for large scale network evacuation planning and management: A literature review. Surv. Oper. Res. Manag. Sci..

[13] Haga Y., Kim T., Mihashi N. (2008). Actual conditions and trends of how regional facilities are used as designated shelters in the Niigata Chuetsu Earthquake-A case study of designated shelters in the former Nagaoka City. Archit. Inst. Jpn. Plan. Pap..

[14] Sasaki K., Katsumata H. (2015). Actual conditions of the temple used in the event of a wide-area disaster and intention to designate emergency evacuation sites and shelters. Archit. Inst. Jpn. Plan. Proc..

[15] Funakoshi Y., Hatayama M. (2016). Identification of shelters in the case of the Kumamoto earthquake and research on wide-area evacuation across municipalities. IPSJ Res. Rep..

[16] Araki Y., Utagawa M., Takada Y., Tsuboi K., Kitago A. (2017). A study on how to deal with evacuees other than designated shelters-A case study of Mashiki Town in the 2016 Kumamoto Earthquake. Reg. Saf. Proc. Soc..

[17] Tsubogawa H., Miura S., Nagasaka T., Nagamatsu S., Ikeda S. (2009). Kashiwazaki City Community and Disaster Response Issues. Major Disaster Survey.

[18] (2009). Miyagi Prefecture, Miyagi Disaster Prevention Education Basic Policy. https://www.pref.miyagi.jp/uploaded/attachment/15509.pdf.

[19] Numada M., Takatsu S., Yamauchi Y., Nakai Y., Meguro K., Ito T., Hiramatsu S., Izuma N., Akatsu Y., Sato K. (2016). Verification of shelter information sharing system COCOA in Ishinomaki comprehensive disaster prevention drill. Prod. Res..

[20] Bando A., Machida C. (2015). Issues related to information sharing in the event of a disaster in local governments-Collaboration between the disaster prevention department and the medical department. JSCE Proc. F6.

[21] Cabinet Office (2015). 2015 White Paper on Disaster Prevention.

[22] Shiraki W., Ishino S., Izumita K., Doi M., Inomo H., Takahashi R. (2019). Proposal of shelter management issues and countermeasures in the event of an unexpected disaster from the perspective of resilience. JSCE Proc. F6.

[23] (2015). Koganei City: Disaster Prevention Plan in Koganei City.

[24] (2015). Rikuzentakata City: Manual of Shelter Management.

